# Leveraging stylometry analysis to identify unique characteristics of peer support user groups in online mental health forums

**DOI:** 10.1038/s41598-023-50490-w

**Published:** 2023-12-27

**Authors:** Yong-Bin Kang, Anthony McCosker, Jane Farmer

**Affiliations:** 1 Australian Research Council (ARC) Centre of Excellence for Automated Decision-Making and Society (ADM+S), Swinburne University of Technology, Hawthorn, VIC 3122 Australia; 2 Social Innovation Research Institute, Swinburne University of Technology, Hawthorn, VIC 3122 Australia

**Keywords:** Psychology, Health care, Public health

## Abstract

Online peer support mental health forums provide an effective and accessible form of support, augmenting scarce clinical and face-to-face assistance. However, to enhance their effectiveness, it is essential to understand the unique characteristics of peer support user groups, and how they participate, contribute and communicate in these forums. This paper proposes and tests a novel approach that leverages stylometry analysis to uncover the unique characteristics of peer support user groups in such forums. Our approach identifies how each group empowers and supports other users, and what distinguishes them from others. The analysis shows that emotion-related words are crucial in identifying and distinguishing user groups based on their writing style. Comparative analysis of emotion expressions across user groups also uncovers the significance of emotional content in these forums in promoting mental well-being. Valued ‘senior contributors’ were more likely than all other groups including trained community guides to use a wide range of both positive and negative emotions in their posts. These findings have significant implications for improving the training of peer-mentors and moderators, scaling forum services, and improving guidelines for emotional expression among peer support users. Our approach presents an objective approach to differentiating the characteristics and communication patterns of valued senior contributors, mentors, and guides, enabling service providers to foster the kinds of communication that supports positive outcomes for distressed users.

## Introduction

The prevalence of mental illness is on the rise worldwide. The COVID-19 pandemic contributed to a global mental health crisis, increasing the number of people seeking mental health treatment^1^, while also shifting support to digital services and telehealth. It is estimated that 3.4 million Australians (17.5%) aged 16 to 85 years sought help from mental health professionals during 2020 and 2021. Among them, 57.4% had a 12-month mental disorder^2^. Over the past few decades, the Internet has permeated most aspects of human life and enabled an easy access to health information online, with 50–70% of internet users searching for answers to specific health-related questions^3^. In Australia, online peer support mental health forums play an increasingly crucial role in enhancing resilience of those with mental illness^4,5^ (e.g., those run in Australia by the charities such as SANE Australia^6^, Beyond Blue^7^, and ReachOut^8^). Their roles have been shown to be highly effective in building social connections, sharing information, guidance and experiences, and offering emotional cares among people with lived experiences of mental illness. In this context, the ‘peer support’ refers to a mutual exchange of emotional and practical support among individuals who identify as peers based on their shared or similar experiences of mental distress^9^. Recently, online peer support forums have become increasingly popular, especially, the COVID-19 pandemic have disrupted conventional mental healthcare and led to a rapid shift to this communication platform. The 2020 SANE Australia annual report^10^ found that the SANE’s forum membership grew by 29.3% with 16,275 registered members in 2019 to 21,041 in 2020; the number of unique visitors increased by 19% compared to 2019; and 151,137 Australians used the forums in 2020 which is an increase of 31% on 2019.

In such forums, diverse peer support user groups (e.g., specialised trained staff, volunteers and untrained peers) work together to create and promote a supportive and safe space to talk and be heard. Often, these groups are moderated by peers or professionals, or a combination of both and can take a variety of forms, such as being organised independently in the community or as part of ongoing mental health care^11^. These groups are comprised of regular and intermittent users, as well as new users. Although essential to the success of online mental health support, the strengths and characteristics of peer support user groups are understudied. Identifying and enhancing these strengths through training and modelling can improve the quality of their support. Many studies have focused on assessing the overall effectiveness of peer support users on improving mental health in general, such as (1) strengthening social connectedness based on shared experiences, (2) and empowering peers through peer support, (3) better coping with day-to-day challenges and stigma reduction, and (4) promoting quality of life, hope and social wellness^9,11–13^. Other studies also conducted on identifying differences in ‘user engagement’ in online mental health communities. Their common goal was to improve practices and policies to promote the productivity, growth and sustainability of online activities of peer support users, as well as reducing unsatisfactory and uneven quality of their word expressions. For example, some studies attempted to measure the importance of ‘highly-engaged peer leaders’ who communicate with many other users by means of ‘post frequency’^14–16^. These studies found that highly-engaged users (i.e., high-posting users) are likely to be important contributors to the effectiveness of online health communities. Another study^17^ tried to identify associations between the users and the posts relevant to topics being discussed (generally a topic is seen as a cluster of words frequently co-occurring together). However, it assumed that posts can be simply divided into binary ‘on-’ and ‘off-’ topics, thus ignoring the important fact that relevance of posts is subjectively determined by users^18^.

One study^18^ sought to show the relationship between users and topics topics by measuring the number of users engaging in discussed topics by associating the posts of each user with those topics. Based on this scheme, that study identified the dominant role of super users (the top 1% of users) and their effect on other users in online mental health forums. Along similar lines, other studies focused on measuring the impact of ‘prolonged participation’ in an online depression community and its positive effect on emotion changes in peer users by examining emotion-related language usages^19^ and clustering of terms from posts in online depression forums^20^. Also, a study found that ‘user participation styles’ (i.e., reading, posting, and responding) affected the benefits of users with depression from online peer support depression community^21^. Another recent study^22^ extended the research on the content and participation of users in online depression forums by observing correlations between posts, the prevailing topics and comment types of users on the posts (e.g., positiveness, length of comments), to aid the development of online interventions for depression.

Previous research has attempted to understand the roles and responsibilities of peer support users in online mental health communities through *indirect* measures, such as extracting discussed topics using topic modelling and analysing associations between such topics and participation patterns. While informative, these methods addressed the characterisation of users based on broad content themes, thereby indirectly capturing users’ traits. However, these approaches have so far failed to capture the unique characteristics of user groups *directly* from their written expressions, leaving the intrinsic ‘nature’ of these groups elusive. In contrast, our study harnesses stylometry analysis, a form of that directly analyses linguistic nuances and characteristics present in users’ written expressions. Furthermore, this identification can also help ensure that peer support user groups are better equipped to promote a supportive and safe environment for all members. Ultimately, this identification can enhance the efficacy of user groups in improving the overall mental well-being and resilience of individuals with mental illnesses. However, complexity and the lack of a systematic approach for identifying, characterising, and comparing the pivotal characteristics of peer support user groups through their online expressions has impeded progress in better understanding their unique nature. To address this gap, this study aims to uncover the distinct characteristics of user groups in online peer support mental health forums by conducting *stylometry analysis* of their forum posts. A credible assumption is that unique characteristics of user groups can be manifested in their writings, which are the primary means of communications between users in online forums. A person’s writing style is commonly considered as unique, much like a personal fingerprint^23^. Stylometry analysis has been proven to be an effective tool in identifying and understanding the writing styles and linguistic patterns of individuals^24^. For example, it has been used to identify psychological distress levels of youth individuals by examining some stylometric features in their narrative writings^25^. *Stylometric features* are characteristics of an individual’s writing style that can be quantified and analysed using computational methods. These features can include various linguistic aspects such as word choice, sentence length, and punctuation usage, among others.

This paper makes two major contributions to the field of online peer support for mental health. Firstly, a new approach is proposed for identifying the unique characteristics of user groups in mental health forums using a popular stylometry analysis method, *John Burrows’ Delta* statistic method (simply the Delta method). This method involves selecting the *n* most frequently used words, referred to as stylometric features, from all texts written by all groups. Our study explores different types of stylometric features, including ‘emotion-related’ words, ‘pronouns’, and ‘unigrams’ and their use by different groups of forum users. Previous research has shown that unigrams are commonly used^24^, while emotion-related words have been found to provide important insights into the emotional states of people experiencing mental health challenges^25^. Likewise, pronouns have often demonstrated importance in understanding emotional states^26^. Specifically, we found that emotion-related words showed higher effectiveness in user group identification. Expanding on the roles of these distinct word types will provide a more comprehensive understanding of their pivotal significance within our research context. Secondly, we conduct a comprehensive comparative analysis of the use of emotion expressions across user groups. In online health communities, attribution of roles such as ’senior contributor’ or ’casual contributor’ provides an important signal to other members, generating trust and facilitating support. This study provides crucial insights into the similarities and differences between user groups and provides a method for identifying users’ roles through their use of emotional expression. This can in turn support training practices and opportunities for senior contributors, moderators and guides.

## Methods

### Data

SANE Australia offers peer support services, training, and counselling for Australians affected by mental illness. The SANE data were collected from two online forums^27^: (1) ‘Lived Experience Forum’ and (2) ‘Friend, Family, and Carers Forum’. The former is a space for people who struggle with complex mental health issues to discuss and seek support for various mental health challenges. This inclusivity enables open and supportive discussions, catering to the broad spectrum of mental health difficulties. The latter is for those who support and care for these individuals. These forums are moderated by mental health practitioners and provide a safe and anonymous place to discuss mental health issues. Through these forums, participants can establish social connections, share feelings and emotions, and assist others with mental illness by exploring positive conversations to their problems. We combined these two forums for our analysis for two reasons: (1) broadening the scope and diversity of our analysis to gain a more comprehensive understanding of online mental health communities, and (2) conducting a joint analysis of both data to unveil shared patterns as well as distinct characteristics among the various user groups present in these communities. For this study, a national sample of SANE forum posts was collected between July 2018 and December 2020. In collaboration with SANE forum managers, 70,179 posts were obtained by 2357 users.

#### Target user groups

This study analysed a total of 70,179 posts by 16 pre-defined groups of users who are based in Australia. The forum management team at SANE establishes a set of user categories and assign these to each user’s profile. The category is visible to others on the forum and helps to distinguish roles, particularly for those who are enlisted to provide peer support (‘community guides’, ‘moderator’, ‘peer support worker’). Community guides, moderators, and peer support workers are more formal roles, and receive training or are involved more directly in forum management. Senior contributors and community elders, on the other hand are categorised as such in recognition of their longstanding involvement and helpful contribution to the forums. The roles and categories are attributed by SANE’s forum managers through subjective decisions based on various factors such as users’ level of participation, personal experiences, the nature of their contributions. Forum managers recognised the importance of role allocation and sought better means of attributing roles and potentially automating this process. We used these categories as a valuable starting point to develop a method for determining clearer and more meaningful distinctions between groups of contributors and to be able to specify their attributes.

The top-5 user groups—‘senior contributor’ (54.1%), ‘community guide’ (24.2%), ‘community elder’ (5.9%), ‘casual contributor’ (3.4%), and ‘contributor’ (3.3%)—accounted for 80% of the total posts. The remaining 20% of posts were contributed by the rest of 11 user groups, including ‘community manager’, ‘new contributor’, ‘moderator’, ‘community builder’, ‘peer ambassador’, ‘peer support worker’, ‘special guest’, ‘bushfire community ambassador’, ‘community manager—peer ambassadors’, ‘administrator’, and ‘trainee moderator’, with their contributions ranging from 0.01% to less than 3%. Our analysis focuses on exploring the top-5 user groups, which made significant contribution to the total posts, as prior studies have shown that highly influential users are often identified by their high posting frequency, and these users are generally highly valued^15,18^. In total, 1289 users (i.e., 55% of all users) belonged these top-5 groups.

As seen in Table 1, ‘senior contributor’ was the most contributing group in terms of posting frequency. The ‘community guide’ group posted 27% of the total posts, while the remaining three groups contributing fewer posts. Interestingly, the top-2 groups, ‘senior contributor’ and ‘community guide’, posted the most, but their individual posts were shorter in word count compared to the other three groups. Notably, ‘casual contributor’ had more than double the number of words per post compared to ‘senior contributor’. Statistical analysis using Mann–Whitney U tests revealed that the word count differences among these groups were significant based on their medians, with all *p* values less than 0.0001.

**Table 1:** Target user groups.

User group	Total number of posts	Mean (sd) post word count	Median word count
Senior contributor	37,999	71 (116)	40
Community guide	16,998	50 (85)	26
Community elder	4147	77 (95)	52
Casual contributor	2405	149 (206)	82
Contributor	2321	117 (116)	59

#### Ethical Approval

This study was approved by Swinburne University Human Research Ethics Committee (SUREC) (R/2019/033). The SUREC committee approved the ethics application on 01/02/2019, responding to the request for consent waiver based on impracticability, low risk and risk mitigation strategies in line with Australia’s National human ethics guidelines, the National Statement on Ethical Conduct in Human Research (2007). All methods and analyses were performed in accordance with the protocol submitted and reviewed by the SUREC ethics committee. We also adhered to the SANE’s ethics and data governance policies throughout, such as establishing a data sharing agreement, anonymising forum posts, and applying a data security protocol.

### Overview of analysis

Our analysis consisted of two main parts. In Part 1, we conducted stylometry analysis to identify the unique characteristics of user groups with exploring various stylometric features. The analysis identified that emotion-related words can effectively predict the writing styles of each user group. In Part 2, we performed a comparative analysis of the distribution of emotion-related words among user groups to quantify the degree of differences in their emotional expressions.

Stylometry analysis is a well-established method that has been widely used for *authorship attribution* based on quantitatively measured linguistic evidence. This method aims to determine the correct author of a new, previously unseen document by analysing specific stylometric features that are extracted from a set of texts with known authorship^28^. By comparing the stylometric features of the new document to those of the known texts, this analysis can provide insights into the likely authorship of the new document. The underlying assumption in stylometry analysis is that individuals have idiosyncratic habits of language usage, resulting in stylistic similarities between texts written by the same person. This analysis quantifies such habits using various stylometric features, such as lexical features (e.g., sentence/line length), syntactic features (e.g., relative frequency of most common words), and content-specific features (e.g., important keywords on specific topics)^29^. These features are then used to quantify the overall similarities between texts written by different authors. Finally, this information is used to identify the most likely author of an unknown text. In our study, we use the Delta method^30^, as it is one of the most prominent methods in use today. It can measure the distance between a text whose authorship that we aim to verify and some other corpus. Specifically, it measures how an unknown text and the texts written by a number of known authors (‘user groups’ in the context of this study) diverge from the average of all of them. In our context, the Delta method is summarised as follows:

1. Create a text collection (called *corpus*) by merging the posts written by *n* ($n=5$) user groups.
2. Find the *k* most frequent words in the corpus and use them as stylometric features. For each of these *k* features, calculate its frequency over the total number of words in each group’s text collection (called *subcorpus*). Then, calculate the mean and the standard deviation of these *n* frequencies and use them as the mean and standard deviation for this feature over the corpus.
3. Standardise these word frequencies (i.e., normalising the frequencies such that, over the whole corpus, the mean for each word is 0 and the standard deviation is 1 (also known as the *z*-score)). For each feature $f_i$ and a subcorpus *C*, calculate a *z*-score describing how far away from the corpus norm the $f_i$’s usage in *C* (the *z*-score for $f_i$): $z_i = \frac{C_i-\mu _i}{\sigma _i}$, where $C_i$ is the frequency of $f_i$ in *C*, $\mu$ denotes the $f_i$’s mean, and $\sigma$ is the $f_i$’s standard deviation.
4. Calculate the same *z*-score for each feature in the unknown text *T* for which we wish to determine authorship.
5. Finally, calculate a delta score $\Delta _C$ comparing *T* with each group’s subcorpus *C*. That is, compute the average of the absolute values of the differences between the *z*-scores for all features between *T* and *C*: $\Delta _c = \sum _{i}\frac{|Z_{(C,i)}-Z_{(T,i)}|}{k}$, where $Z_{(C,i)}$ is the *z*-score for feature $f_i$ in candidate *C*, and $Z_{(T,i)}$ is the *z*-score for $f_i$ in *T*.
6. The most likely user group is the group for whom the delta score between the group’s subcorpus and *T* is the lowest.

The Delta method was developed to compare texts by identifying unique linguistic patterns. Initially, the method aimed to reduce a large pool of authors to a smaller set for in-depth statistical analysis. Over time, scholars extended Delta’s application beyond epic poems and the English language, demonstrating its effectiveness in diverse text genres and languages^31^. Delta has proven to be one of the most robust intertextual distance measures between two texts^32^, showing promising applicability in datasets characterised by individual writing styles and varied linguistic patterns^32,33^. We utilised the Delta method to decipher nuanced linguistic patterns using different features, which aligns with our objective to precisely identify distinct user groups’ characteristics and emotional expressions within online mental health communities.

## Part 1: Identification of characteristics of user groups

### Methods

In Step 2 of the Delta method, we needed to identify two critical pieces of information. Firstly, we determined the most useful type of frequent words to accurately characterise each user group and distinguish them from one another. Once this was identified, secondly, we proceeded to find an appropriate value for *k*, which represents the number of most frequent words. Thus, we explored a combination of both *content-specific* features and *syntactic* features to identify stylometric features. Firstly, as content-specific features, we investigated four different word types: (1) tokens (unigram), (2) emotion-related words, (3) tokens+pronouns, and (4) emotion-related words+pronouns. Our goal was to determine the best type of words for maximising the identification of user groups from their writings. Tokens refer to a comprehensive set of the most frequently occurring unigrams derived from the corpus. To enhance the semantic relevance of the tokens, common stopwords, which are typically considered to have minimal impact on sentence meaning, have been excluded from the collection. Tokens have been popularly used in stylometry analysis^24^. We also measured the impact of *emotion-related words* that have been found to be significant in identifying different levels of mental illness^25^. An emotion-related word is the word that is associated with one or more than particular emotions, such as happiness, sadness, anger or fear. Further, we explored the combined effects of tokens and emotion-related words with pronouns (i.e., tokens+pronouns and emotion-related words+pronouns), as previous research found that pronouns (e.g., I, You, We, etc) often offer crucial insights into emotional and relational states in mental health^26^.

Secondly, as syntactic features, we explored different numbers for *k* in Step 2 of the Delta method. Our goal was to determine an optimal number of *k* that contributes to the most accurate identification of user groups. Previous research suggested that generally 50–100 most common words can be effective in reliably distinguishing authors^31^. However, an optimal value of *k* may vary depending on factors such as the length of the text, the number of authors, and the writing style of the authors. A larger value of *k* may result in a more detailed analysis but also increase the risk of overfitting, while a smaller value may lead to less accurate but more computationally efficient. Thus, rather than using an adhoc number, we tested a range of values for *k*, between 50 and 500 by an increment of 50, to find its optimal value. We used the Fast Stylometry library^34^ to implement the Delta method. To extract tokens and pronouns from all posts, we utilised a well-known NLP library, spaCy^35^. To identify emotion-related words, we leveraged the NRCLex dictionary that comprises English words associated with eight primary emotions^36^. These emotional associations were manually annotated through crowdsourcing. It enabled to extract these emotions and their associated emotion-related words from the posts: four positive emotions include *anticipation*, *trust*, *surprise*, and *joy*, while the other four negative emotions include *fear*, *anger*, *sadness*, and *disgust*.

Moreover, our aim was not only to develop an identification model for user groups using the Delta method, but also to validate the accuracy of the model. To achieve this, we randomly divided the total posts into two sets: 80% of the posts were used as training data, while the remaining 20% were used as test data. This random data splitting enabled an evaluation of the model’s performance on an independent dataset, which helps in assessing its generalisability beyond the training data, aiming to address overfitting, a common challenge in machine learning, to ensure the model’s performance on new data.

Furthermore, to improve our interpretation of the identification model, we conducted two additional steps. Firstly, we performed a calibration step to improve the interpretability and accuracy of the result. The Delta method produces statistics (such as delta scores in Step 5) that measure the distances between user groups based on their writing styles represented by stylometric features. However, these statistics alone can be difficult to interpret and may not directly reflect the probability that two pieces of text were written by the same group. By calibrating the model, we transformed these statistics into more meaningful and accurate representations of the probability that two pieces of text were written by the same group. To achieve this, we determined the commonest Delta statistics that indicate the same authorship among the known groups in the training data. This involved calculating the Delta statistics for each pairwise combination of the posts written by different user groups in the training data. The resulting values were used to construct a calibration curve that maps the Delta statistics to probabilities.

To calibrate the model, we used a logistic regression classifier that is a common method due to its simplicity, flexibility, high performance and fast computation^37^. Specifically, we used the LogisticRegression classifier in the scikit-learn library in the Fast Stylometry library, configured with class_weight$=$‘balanced’. This parameter was chosen to address class imbalance concerns in the data. By adjusting the class weights inversely proportional to the class frequencies, this setting manages imbalanced classes during the training process. Once the calibration step is done, our second step involves generating an *Area Under the Curve* (AUC) score from the probabilities produced in the previous step. The AUC score is a widely used metric for evaluating the quality of a machine learning model, representing the area under the *Receiver Operating Characteristic* (ROC) curve. The ROC curve plots the *true positive rate* (TPR) against the *false positive rate* (FPR) of a classifier. To generate the AUC score, we first calculated the TPR and FPR of the logistic regression classifier using the predictions on the test data. The ROC curve is then generated by plotting the TPR against the FPR, and the AUC score is calculated as the area under the curve. The AUC score provides a single number that summarises the overall performance of the classifier, which in turn summarises our Delta model. A perfect classifier would have an AUC score of 1.0, while a completely random classifier would have an AUC score of 0.5. Generally, an AUC score of 0.7–0.8 is considered acceptable, while a score of 0.9 or higher is considered excellent. Therefore, by conducting the calibration step and generating an AUC score, we were able to validate the accuracy of our group identification model and improve our interpretation of the results.

Finally, we used the AUC scores computed on the test data to identify the most effective type of stylometric features and an optimal value for *k*. By leveraging these features, we were able to uncover the distinctive characteristics that set each user group apart from the others, providing a unique means of identifying and distinguishing them.

### Results

**Figure 1:**
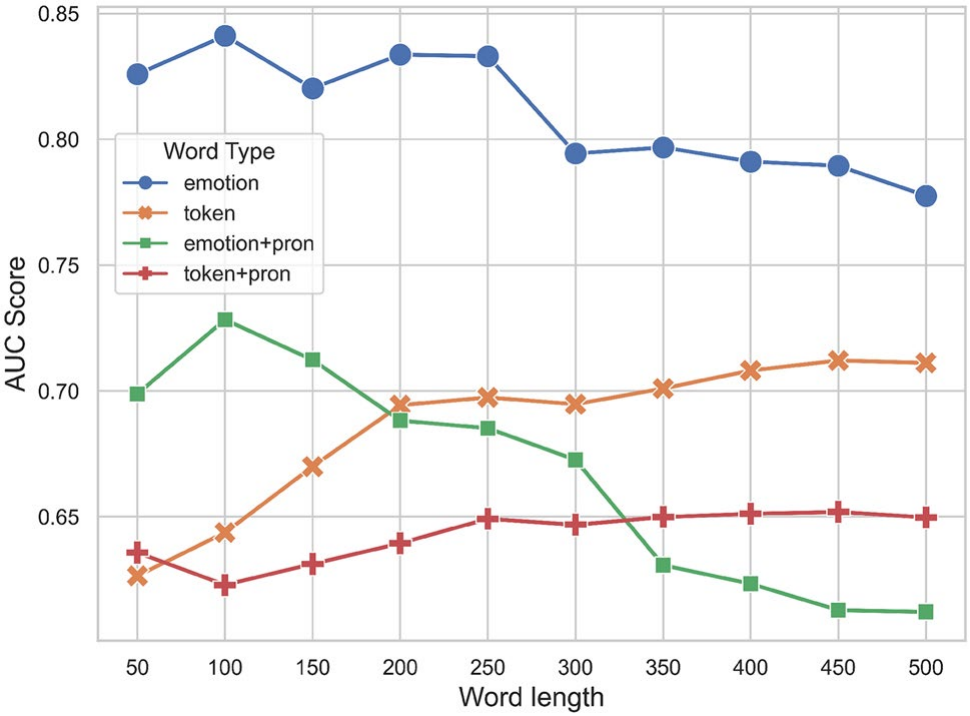
Comparison of the user group prediction performance using the four types of stylometric features with a range of the most frequent words in the stylometry analysis.

The results of the 5 user group identification models, using the four types of stylometric features, are presented in Fig. 1. These types include tokens, emotion-related words, tokens+pronouns, and emotion-related words+pronouns, denoted by ‘token’, ‘emotion’, ‘token+pron’, and ‘emotion+pron’ respectively in the legend. The x-axis represents the number of most frequent words, while the y-axis shows the AUC scores that represent the performance measures of the models trained on known groups in the training data for predicting correct groups in the test data (unknown posts), where each model was built and evaluated using each of the four feature types. A key observation is that the model using the ‘emotion’ feature type consistently outperformed all the other models across all different numbers of the most frequent words. Adding ‘pronouns’ to the ‘token’ and ‘emotion’ features did not improve the performance of the models, even resulting in a negative impact. The AUC score of the best model increases from around 0.83 for the 50 most frequent words to a peak of 0.84 at the 100 most frequent words, gradually declining to around 0.78 for the 500 most frequent words. This indicates that using a larger number of the most frequent words may not improve the model’s performance. As seen, we identified 100 as an optimal number for *k* for the best model. Overall, the model that includes both tokens and pronouns performed the worse, with AUC scores ranging from around 0.62 to 0.65.

To gain insights into the performance of the best model that uses the ‘emotion’ feature type with the 100 most frequent words, we present its Delta statistics and calibrated probabilities in Tables 2 and 3, respectively. Table 2 shows the average Delta statistics that measure the distances between the posts written by a group in the training data (rows) and a group in the test data (columns). The value with the lowest distance on each row, indicating the closest match between the writing styles of a group in the training data and a group in the test data, is highlighted in bold. However, interpreting this matrix can be challenging, and it would be more intuitive to have a probability value that represents the likelihood of each group in the test data being the same as the one in the training data. Table 3 shows the calibrated probabilities obtained by averaging the Delta statistics shown in Table 2. Each value shows the average of the calibrated probabilities that the posts of a group in the test data were written by a group in the training data. Each value in bold represents the highest one among the probabilities between a group in the training data and all groups in the test data. As shown in Table 3, on average, the model’s probabilities for all 5 user groups are very high, ranging from 0.85 to 0.91. For instance, we can interpret that there is a 91% probability that the posts in the test data attributed to ‘contributor’ were actually written by the same group in the training data.

**Table 2:** The Delta statistics of the model using the ‘emotion’ feature type with the 100 most frequent words. Each group on a row indicates its posts in the training data, while each group on a column denotes its posts in the test data.

Training/testing	Senior contributor	Community guide	Community elder	Casual contributor	Contributor
Senior contributor	**1**.**40**	1.54	1.56	1.89	1.52
Community guide	1.75	**1**.**23**	1.73	2.33	1.90
Community elder	1.71	1.60	**1**.**35**	2.03	1.75
Casual contributor	1.82	2.02	1.96	**1**.**44**	1.48
Contributor	1.73	1.81	1.73	1.58	**1**.**46**

**Table 3:** The probabilities that each group in the testing data (a column) is written by each user group in the training data (a row). The probabilities were calculated by transforming the Delta statics into the probabilities via calibration.

Training/testing	Senior contributor	Community guide	Community elder	Casual contributor	Contributor
Senior contributor	**0**.**85**	0.75	0.79	0.60	0.86
Community guide	0.61	**0**.**88**	0.68	0.28	0.68
Community elder	0.69	0.73	**0**.**90**	0.47	0.70
Casual contributor	0.61	0.38	0.51	**0**.**90**	0.85
Contributor	0.71	0.62	0.75	0.82	**0**.**91**

## Part 2: Comparison of emotion expression across user groups

### Methods

Following our analysis in Part 1, we found that emotion-related words were the most significant distinguishing feature, differentiating the writing styles of the 5 user groups. In Part 2, our goal was to dive deeper into the unique emotional expressions of each user group. This in-depth examination aimed to uncover nuanced insights into how the distinct groups express and manifest emotions within their writing. To achieve this, firstly, we examined the frequency distributions of emotion-related words and explored the specific emotional lexicon and expressions characteristic of each user group. To avoid the influence of outliers, we did not use the average frequencies of these words for comparison. Secondly, we used chi-square tests to compare the distributions between all 5 groups in terms of their collective use of emotion-related words as well as binary emotion concepts (i.e., positive and negative concepts), to detect any significant differences. Thirdly, we investigated differences between the ‘senior contributor’ (the most contributing group as seen in Table 1) and the other groups in their use of emotion-related words. To do this, we compared the odds of each emotion-related word between the ‘senior contributor’ and each of the other groups to determine whether the odds ratio of using the word in two groups is equal to one. We used chi-square tests to measure the significance of the differences in the measured odd ratios. By doing so, we were able to understand the specific emotion expression patterns of the ‘senior contributor’ group, in comparison with the other groups. Finally, to aid in visualising the patterns and distribution of emotion expressions among the groups, we created a radar chart. The chart allowed us to quickly identify similarities and differences in such expressions among the groups. This also helped us to pinpoint groups that were particularly high or low in certain emotions, allowing for a more nuanced understanding of the emotion expressions among the groups.

When conducting multiple comparisons using the above chi-square tests, the probability of making a type I error (i.e., rejecting the null hypothesis when it is true) increases, which is known as the multiple comparisons bias. To avoid this, we applied multiple correction using the well-known the Benjamini-Hochberg procedure, which involves adjusting the *p* values to reduce the likelihood of falsely rejecting the null hypothesis and increase the reliability of the results. This ensured that our results were more robust and accurate, and that significant differences between the user groups were not due to chance.

### Results

To understand the dominant emotion expressions of all 5 user groups, we generated a list of the 100 most frequently occurring emotion-related words in the total posts combining the training and test data used in the Part 1 analysis. In Part 2 analysis was conducted based on these total posts. The list is presented in Table 4. The most commonly used word is ‘time’, which is linked to the positive emotion, ‘anticipation’, based on its frequent contextual usage in a large text corpus, according to the NRCLex. The second and third most frequent words, ‘good’ and ‘hope’, convey all four positive emotions pre-defined by NRCLex: ‘anticipation’, ‘joy’, ‘surprise’, and ‘trust’. Interestingly, all top-5 words are associated to positive emotions; and among these 100 words, over 70% of the words indicate positive emotions, while the remaining words indicate negative emotions.

**Table 4:** Top 100 common emotion words from the total posts.

Rank	Word	Freq	Rank	Word	Freq	Rank	Word	Freq	Rank	Word	Freq
1	Time	12,067	26	Kind	1612	51	Eat	1094	76	Diagnosis	798
2	Good	12,012	27	Learn	1573	52	Illness	1076	77	Birthday	798
3	Hope	9060	28	Problem	1534	53	Sense	1075	78	Advice	783
4	Love	4892	29	Pretty	1529	54	Wait	1064	79	Contact	777
5	Friend	4150	30	Word	1510	55	Rest	1056	80	Full	754
6	Talk	3924	31	Mum	1482	56	Agree	1047	81	Symptom	753
7	Start	3567	32	Plan	1455	57	Forward	1047	82	Visit	750
8	Long	3547	33	Daughter	1431	58	Real	1034	83	Pay	739
9	Bad	3262	34	Pain	1409	59	Small	1019	84	Community	734
10	Happen	2826	35	Lose	1390	60	Wrong	1001	85	Late	731
11	Leave	2638	36	Depression	1376	61	Reason	1000	86	Fear	727
12	Change	2398	37	Manage	1368	62	Show	998	87	Offer	720
13	Happy	2177	38	Difficult	1366	63	Doctor	997	88	Catch	715
14	Thought	2073	39	Enjoy	1327	64	Tough	986	89	Join	715
15	Anxiety	1990	40	Guess	1324	65	Hurt	963	90	Food	707
16	Hug	1964	41	Break	1289	66	School	947	91	Hate	698
17	Feeling	1924	42	Helpful	1276	67	Beautiful	945	92	Treat	686
18	Hospital	1914	43	Important	1264	68	Young	920	93	Worth	679
19	Struggle	1912	44	Don	1262	69	Stress	907	94	Include	679
20	Share	1869	45	Lovely	1173	70	Worry	870	95	Disorder	672
21	Glad	1861	46	Mother	1173	71	Case	866	96	Fall	666
22	Job	1806	47	Watch	1151	72	Abuse	864	97	Money	660
23	Deal	1754	48	Question	1148	73	Continue	852	98	System	655
24	Tomorrow	1691	49	Partner	1107	74	Tired	842	99	Suffer	643
25	Child	1648	50	Safe	1107	75	Wonderful	839	100	Study	643

Table 5 shows examples of emotion-related words and their associated emotions found in some posts. As observed, emotion-related words can be triggered by a wide range of topics, such as physical health (e.g., infection, sick), mental health (e.g., struggle), and education (e.g., university studies). The use of such words shows that individuals are not only sharing factual information but also providing an emotional reaction and response to other’s posts. Recall that NRCLex associates words with emotions based on how they are typically used in contexts, using a large corpus of text to identify the most common emotional associations for each word.

**Table 5:** Examples of emotions expressed in posts (deidentified words are marked as ‘xxx’).

Posts	Emotion-related words and their emotions
Thanks xxx I hope you feel better soon too. xxx infection, maybe chest too (I hope not). A course of antibiotics from locum Dr.Medical Certificate, & had to call in sick (not work) for tomorrow. xxx	hope: [anticipation, joy, surprise, trust], infection: [fear], sick: [disgust, sadness], tomorrow: [anticipation]
Hello xxx sorry to hear of xxx and chest infection...xxx can be very painful....does the towel over the head inhaling epsom salts in hot water help? some people use vicks vaparub...chest infection...antibiotics or penicillin? good that you are not going to work...your body needs you to rest to start to heal...keep in touch with us here...when the flipping internet is working I will respond when I am on here.	infection: [fear], painful: [anger, disgust, fear, sadness], hot: [anger], good: [anticipation, joy, surprise, trust], start: [anticipation], heal: [joy, trust]
xxx Thank you for your reply.. You are doing the right thing...making the choice to release some of that stuff that you struggle with on a daily basis by venting on here..keep it up...gradually you will start to notice small moments of relief..you.	struggle: [anger, fear, negative, sadness], daily: [anticipation], start: [anticipation]
good morning xxx Yes ...spot on...some are volunteers with lived experience...some perhaps no lived experience..counselling trained....The fact that someone has lived experience does not make them a good listener...that is part of their character Some are very good listeners...reassuring when not knowing what to say...even reassuring that not taking up too much time...always ring again and let them know how you felt as this reinforces your own value as well as provide vital feedback...hoping that rest and antibiotics doing there work will lead you to recovery soon..take care..	good: [anticipation, joy, surprise, trust], volunteers: [trust], fact: [trust], time: [anticipation], provide: [trust], hoping: [anticipation, joy, surprise, trust], recovery: [anticipation]
Thanks xxx It was a good day xxx. Settled in with our art supplies delivered and lockers assigned, and have started classes already. The house didn’t fall down without me here, and, despite having half his sleep happening at night and the other half happening during the day, made it to his two classes, after being absent for weeks at the end of last semester. xxx has an appointment on Wednesday to start her uni studies off again, hopefully. Everything is still a bit wonky, but we are on the road at least.	good: [anticipation, joy, surprise, trust], art: [anticipation, joy, sadness, surprise], supplies: [anticipation], fall: [sadness], absent: [sadness], happening: [anticipation], start: [anticipation]

Table 6 presents the count (proportion) of emotion-related words used in the total posts by all 5 user groups. Each proportion represents the total count of an emotion-related word over the total number of words in the post collection written by each group. We observe that ‘trust’ is the most dominant emotion expressed by all user groups. This may indicate that trust is a fundamental component of mental health support forums, and its expression through related emotional language is a good indicator of a positive forum environment. Also, the high frequency of ‘trust’ across each user group shows that it underpins the mental health forum as a trustworthy and supportive source of help. Note that the three dominant groups (in terms of post frequency), ‘senior contributor’, ‘community guide’ and ‘community elder’, shared a similar distribution of emotions, primarily using the positive emotions of ‘trust’, ‘anticipation’ and ‘joy’. Both ‘casual contributor’ and ‘contributor’ used language expressing ‘sadness’ and ‘fear’ in addition to ‘trust’ and ‘anticipation’, more regularly than the other groups. Moreover, some emotions such as ‘surprise’ and ‘disgust’ were less frequently used by all groups. Further, the negative emotion ’disgust’ was least used among all groups.

**Table 6:** The count (proportion) of emotion-related words from the total posts by the user groups.

User group	Fear	Anger	Sadness	Disgust	Anticipation	Trust	Surprise	Joy
Senior contributor	79,364 (3.3)	54,634 (2.2)	84,934 (3.5)	37,542 (1.5)	124,842 (5.1)	129,808 (5.3)	60,870 (2.5)	109,566 (4.5)
Community guide	18,734 (2.5)	12,708 (1.7)	19,408 (2.6)	8,276 (1.1)	36,600 (4.8)	39,318 (5.2)	18,586 (2.5)	34,576 (4.6)
Community elder	9236 (3.2)	6148 (2.2)	9386 (3.3)	4,152 (1.5)	14,536 (5.1)	15,666 (5.5)	6954 (2.4)	13,190 (4.6)
Casual contributor	14,998 (4.6)	10,270 (3.1)	15,126 (4.6)	6234 (1.9)	14,910 (4.5)	16,458 (5.0)	6,132 (1.9)	11,634 (3.5)
Contributor	10,186 (4.1)	6706 (2.7)	9,796 (3.9)	4372 (1.8)	11,760 (4.7)	13,108 (5.3)	5038 (2.0)	9626 (3.9)
Average	26,503 (3.5)	18,093 (2.4)	27,730 (3.6)	12,115 (1.6)	40,529 (4.9)	42,871 (5.3)	19,516 (2.3)	35,718 (4.2)

Table 7 presents the distribution of negative and positive emotions across the user groups. On average, all groups expressed positive emotions more frequently (16.6%) than negative emotions (11.3%). The ‘community elder’, ‘senior contributor’, and ‘community guide’ groups had a higher proportion of positive emotions, at 17.6%, 17.4%, and 17.1%, respectively, compared to ‘casual contributor’ and ‘contributor’. The ‘casual contributor’ group had the highest proportion of negative emotions at 14.2%. It is interesting to note that the proportion difference between positive and negative emotions varies among the groups. The three groups, ‘senior contributor’, ‘community guide’ and ‘community elder’, showed a difference of almost 7%, while the difference in the other groups was only about 1-3%. Furthermore, the positive to negative emotion proportions for different user groups is derived, exhibiting distinctive variations: ‘senior contributor’ (1.66:1), ‘community guide’ (2.18:1), ‘community elder’ (1.74:1), ‘casual contributor’ (1.05:1), and ‘contributor’ (1.27:1). These figures help us understand the emotional distribution across the user groups, where ‘community guide’ showed a higher proportion of positive emotions compared to the other groups.

**Table 7:** The count (proportion) of positive and negative emotion-related words from the total posts by the user groups.

User group	Positive emotion	Negative emotion
Senior contributor	425,086 (17.4)	256,474 (10.5)
Community guide	129,080 (17.1)	59,126 (7.8)
Community elder	50,346 (17.6)	28,922 (10.1)
Casual contributor	49,134 (15.0)	46,628 (14.2)
Contributor	39,532 (15.9)	31,060 (12.5)
Average	138,635 (16.6)	84,442 (11.3)

Table 8 presents the results of our chi-square analysis with multiple comparisons correction for emotion expressions between the user groups. The analysis compared pairs of two user groups and showed the chi-square statistic and the corresponding *p* value for each comparison. Examining that all *p* values are less than 0.005 indicates that the observed differences in emotion expressions between the groups were statistically significant. These results suggest that different user groups express emotions differently in their online contributions. Specifically, the strongest difference was observed between ‘community guide’ and ‘casual contributor’, with a chi-square value of 8460.31, showing the difference in emotion expressions between these two groups was highly significant. In addition, ‘senior contributor’ and ‘casual contributor’ also showed a strong difference in emotion expressions, with a chi-square value of 4687.97. Interestingly, this chi-square analysis also revealed significant differences in emotion expressions between user groups that are similar in their use of emotion-related words. For example, the ‘senior contributor’ and ‘community elder’ groups showed a significant difference in emotion expressions, with a chi-square value of 72.70 (a relatively small value). This suggests that even among senior contributors and community elders, there are differences in how they express emotions in their contributions. This knowledge could be useful for understanding communication and interaction among users, as well as for identifying potential emotional support needs within different user groups.

**Table 8:** Chi-square analysis for emotion expressions between user groups from the total posts.

Compared user groups	Chi-square	*p* value
(Senior contributor, community guide)	2564.09	< 0.0001
(Senior contributor, community elder)	72.70	< 0.0001
(Senior contributor, casual contributor)	4687.97	< 0.0001
(Senior contributor, contributor)	1324.68	< 0.0001
(Community guide, community elder)	674.39	< 0.0001
(Community guide, casual contributor)	8460.31	< 0.0001
(Community guide, contributor)	3799.59	< 0.0001
(Community elder, casual contributor)	2810.48	< 0.0001
(Community elder, contributor)	969.4	< 0.0001
(Casual contributor, contributor)	383.37	< 0.0001

Furthermore, we conducted a comparison analysis between the most contributing group, ‘senior contributor’, and the other groups by computing odds ratios. This allowed us to understand the likelihood of a particular emotion being expressed in this group compared to the other groups. The 95% confidence interval (CI) indicates the precision or uncertainty of the odds ratio estimate, with a range of values likely to contain the true odds ratio with 95% confidence. If the CI includes 1, there is no significant difference in the odds of expressing an emotion between two groups. Otherwise, the difference is significant. We used the chi-square test to test the hypothesis that the odds ratio equals 1, and a small *p* value suggests a statistically significant difference. Taken together, the odds ratio, 95% CI, and chi-square test give a comprehensive picture of the strength and significance of the association between two groups in expressing a particular emotion.

**Table 9:** Odds ratios and chi-square analyses for the most contributing group ‘senior contributor’ versus the other groups in terms of emotion expressions from the total posts.

Emotion concept	Compared user group	Odds ratio	95% CI	Chi-square	*p* value
Fear	Community guide	1.32	1.30–1.34	1164.92	< 0.0001
	Community elder	1.01	0.99–1.03	0.41	0.5233
	Casual contributor	0.70	0.69–0.71	1529.97	< 0.0001
	Contributor	0.79	0.77–0.80	501.11	< 0.0001
Anger	Community guide	1.34	1.31–1.37	872.41	< 0.0001
	Community elder	1.04	1.01–1.07	9.27	0.0023
	Casual contributor	0.71	0.69–0.72	1004.77	< 0.0001
	Contributor	0.83	0.80–0.85	213.24	< 0.0001
Sadness	Community guide	1.37	1.35–1.39	1526.92	< 0.0001
	Community elder	1.06	1.04–1.09	30.1	< 0.0001
	Casual contributor	0.75	0.73–0.76	1061.03	< 0.0001
	Contributor	0.88	0.86–0.90	140.76	< 0.0001
Disgust	Community guide	1.41	1.38–1.45	805.39	< 0.0001
	Community elder	1.06	1.03–1.10	12.7	0.0004
	Casual contributor	0.81	0.79–0.83	243.46	< 0.0001
	Contributor	0.87	0.85–0.90	71.45	< 0.0001
Anticipation	Community guide	1.06	1.05–1.07	91.52	< 0.0001
	Community elder	1.01	0.99–1.02	0.56	0.4544
	Casual contributor	1.13	1.11–1.15	196.43	< 0.0001
	Contributor	1.09	1.06–1.11	69.25	< 0.0001
Trust	Community guide	1.02	1.01–1.04	16.53	< 0.0001
	Community elder	0.97	0.95–0.99	12.83	0.0003
	Casual contributor	1.06	1.05–1.08	52.78	< 0.0001
	Contributor	1.01	0.99–1.03	0.95	0.3309
Surprise	Community guide	1.02	1.00–1.03	3.17	0.075
	Community elder	1.03	1.00–1.05	4.13	0.0422
	Casual contributor	1.34	1.31–1.38	478.37	< 0.0001
	Contributor	1.24	1.20–1.27	206.25	< 0.0001
Joy	Community guide	0.98	0.97–0.99	9.15	0.0025
	Community elder	0.97	0.95–0.99	8.96	0.0028
	Casual contributor	1.28	1.25–1.30	614.63	< 0.0001
	Contributor	1.17	1.14–1.19	202.9	< 0.0001

Table 9 presents the odds ratio comparisons between ‘senior contributor’ and the other four groups regarding the expressions of the 8 emotions. The table shows that ‘senior contributor’ used all four negative emotions (i.e., ‘fear’, ‘anger’, ‘sadness’, and ‘disgust’) less frequently than both ‘casual contributor’ and ‘contributor’, while ‘senior contributor’ used all four positive emotions (i.e., ‘anticipation’, ‘trust’, and ‘surprise’, and ‘joy’) more frequently than them. These differences were found to be statistically significant, except for ‘trust’ when comparing ‘senior contributor’ with ‘contributor’, where the *p* value was 0.3309. Comparing with the second most contributing group ‘community guide’, the odds ratios revealed that ‘senior contributor’ used all four negative emotions and three positive emotions (i.e., ‘anticipation’, ‘trust’, and ‘surprise’) more frequently than ‘community guide’. Members of the forum who are ‘senior contributors’ are nominated as such primarily through their ongoing supportive involvement, whereas ‘community guide’ and similar roles are more likely to be trained or to have taken on a formal support role. All differences were statistically significant. Finally, when comparing with the third most contributing group ‘community elder’, ‘senior contributor’ had a similar strength in expressing both negative and positive emotions. The results showed that the ‘senior contributor’ group has different emotional expression patterns in their writing than the other groups.

**Figure 2:**
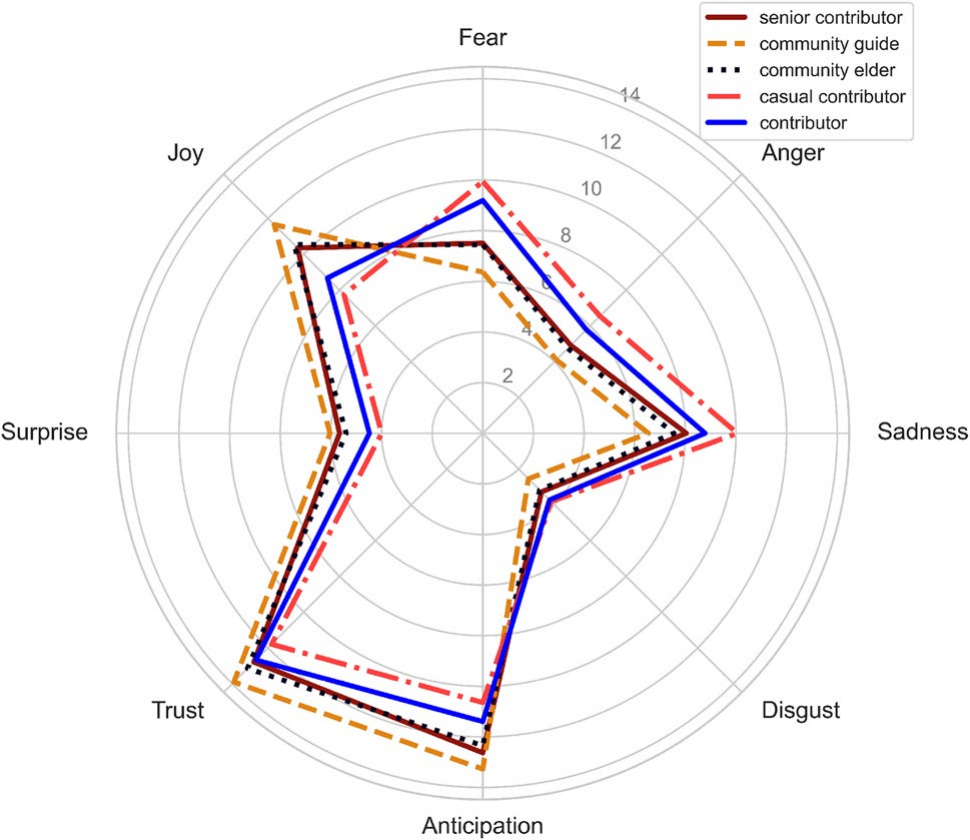
Comparison of the use of the 8 emotions across all 5 user groups.

As the final part of the Part 2 analysis, we present the radar chart in Fig. 2 that represents the emotional profile of the 5 groups. It shows the 8 emotions, with the scores ranging from 0 to 14. The score for each emotion was calculated by averaging the number of the emotion-related words, corresponding that emotion, per post from the posts written by each user group. This method provides a quantitative measure of the relative frequency of each emotion over the posts written by each group. It is observed that ‘anticipation’, ‘trust’, and ‘joy’ were the highest-scoring emotions across all groups, indicating that these emotions are overall well-represented by them. The two groups, ‘casual contributor’ and ‘contributor’, were more likely to score high on ‘fear’, ‘sadness’, and ‘anger’ compared to other groups. The ‘senior contributor’ group had relatively high scores for ‘anticipation’, ‘joy’ and ‘trust’ but low scores for all negative emotions. The ‘community guide’ group had the highest score for all positive emotions among all groups. As we discuss further below, the low level of ‘disgust’ related emotional language suggests that the emotional language used helps to sustain a destigmatising environment as much as a supportive one.

## Discussion

In the Part 1 analysis, firstly, we demonstrated the effectiveness of including emotion-related words in developing a user group identification model using stylometry analysis. This suggests that emotions play a crucial role in differentiating the writing styles of user groups. However, the addition of pronouns did not improve the model’s performance, suggesting their limited informativeness in differentiating writing styles in an online mental health community.

Secondly, we found a smaller number of the most common words may be sufficient for accurately identifying user groups based on their writing style.

Thirdly, we showed that the Delta statistics and calibrated probabilities are valuable metrics for assessing the performance of different models. These measures provide insight into the distances between different writing styles and the probability that two pieces of text were written by the same group. Calibrating the Delta statistics improved the interpretability and accuracy of the results. The high probabilities achieved by the best model across all 5 user groups, ranging from 0.85 to 0.91, indicate a high chance that the posts in the test data were written by the same group in the training data.

Overall, the findings in Part 1 underscore the potential value of emotional content in a stylometry analysis for identifying and distinguishing user groups from one another in the community. Hence, this method can help forum service providers better identify distinct groups of users more accurately, a process that usually occurs through intuitive decisions.

In the Part 2 analysis, firstly, we presented a list of the top-100 emotion-related words used in the posts. We showed that positive emotions dominated emotional expressions in user-generated content. However, the presence of negative emotion-related words, such as ‘depression’, ‘pain’, and ‘stress’, highlighted the potential of identifying users experiencing mental health issues and providing them with appropriate support. This list may also serve as a reference for future studies in emotional analysis of user-generated content.

Secondly, through Table 5, we found that the associations between specific words and complex emotions revealed the intricate nature of human emotions. For instance, the word ‘hope’ was associated with the emotions of ‘anticipation’, ‘joy’, ‘surprise’, and ‘trust’. This indicates that individuals experiences a mix of emotions when expressing hope.

Thirdly, the emotion frequency statistics, presented in Tables 6 and 7, provided significant implications for understanding how online communities function and how user groups interact with each other. The prevalence of positive emotions such as ‘trust’, ‘anticipation’, and ‘joy’ among the dominant groups is particularly noteworthy, as research has shown that positive emotional experiences have been linked to improved mental health outcomes^38^. The absence of emotional words related to ‘disgust’ highlights the predominance and importance of destigmatising language. These results indicate that different user groups have distinct emotion expression patterns, and these patterns are not due to chance. Considering such patterns, it is also worth exploring the influence of factors such as seniority or training, particularly for groups in SANE, to understand their potential roles. Further, research on this topic could provide insights into how to promote positive emotional expression and support within mental health online communities.

Fourthly, the odds ratio comparison results imply that the most contributing group ‘senior contributor’ generally expresses positive emotions more frequently and negative emotions less frequently than the other groups, except for ‘community guide’ and ‘community elder’. We also found that ‘senior contributor’ is most likely to post positively along with ‘community guide’ (second most contributing group) and ‘community elder’ (the third most contributing group). It is also worth noting that the ‘senior contributor’ group was more likely to express the full range of positive and negative emotions than others, showing greater diversity in the way they post. This suggest that this groups may be more involved in conveying encouragement, strength, and hope, compared to the other groups. However, further investigation is necessary to understand why this pattern exists, including whether it is a result of natural tendencies or training provided by SANE, or a combination of both. These findings can help the community managers to identify and provide necessary support and resources to ‘senior contributor’ to continue their valuable contributions to the community.

Finally, the radar chart visualised the emotional profile of the user groups, highlighting similarities and differences in the emotional expression per post across the groups. Three emotions, ‘anticipation’, ‘trust’ and ‘joy’, were particularly salient in the data. The least contributing two groups, ‘casual contributor’ and ‘contributor’, were more likely to express negative emotions in their posts, which could be due to a range of factors such as the topics they are discussing or the nature of their interactions with other members. The distinctiveness of the ‘community guide’ group was that it has the highest score for all positive emotions per post among all groups. This suggests that this group may be trained or position themselves explicitly to create and maintain a positive and open-minded environment, which achieves vital community-building outcomes. This is a crucial observation, particularly in light of the potential for emotion contagion on online platforms. In fact, previous research has shown that positive posting can lead to further positive posting and vice versa^39^, which may be consistent with our findings. It is possible that the ’community guide’ group’s positive attitude is a result of a specific approach to spread positive emotion on the forum or an emergent outcome of encouraging or training high-ranked posters to be positive. Further research and discussion on this topic could provide deeper understanding of the role of emotion contagion in online communities.

In summary, this study provides valuable insights into the emotional dynamics of online mental health communities and forums. These insights can be used to better understand the emotional experiences of community members and to develop effective strategies for managing and moderating emotional expression within these groups. Our findings could also be leveraged to strengthen the training of peer-mentors and moderators, which is crucial for scaling forum services and improving outcomes for users. By identifying the unique characteristics and communication patterns of peer support user groups, our approach can be used to develop targeted training programs that address specific needs and challenges faced by these groups. Furthermore, as the field of digital mental health care continues to grow, the development of chatbots and other automated systems is becoming increasingly common. To develop effective models and training data sets, it is vital to have a thorough understanding of the features, characteristics and approach of valued senior contributors, mentors and guides. Our approach could enhance a more objective way to identify these factors, particularly when used in conjunction with co-design methods. Developers could also create more targeted and effective chatbots and other automated systems that are better equipped to meet the needs of users.

One potential limitation of this study is that it is based on a specific online mental health community, and the results may not be fully generalisable to other similar platforms. Emotion-related words used in the analysis were selected based on a pre-existing list, potentially missing other emotional words not included in the list. In addition, our study primarily focused on analysing the collective characteristics of user groups rather than exploring the characteristics of individual users or super users within each group. Exploring the distinctiveness of these users and their potential influences could provide valuable insights for future studies. Moreover, our word-based analysis might overlook sentence context, prompting the need to investigate methods for incorporating contextual understanding to enhance our model. Moving forward, incorporating user profiles and interactions alongside text-based forum data could provide a more comprehensive view of mental health communities, potentially improving user group identification models. Investigating how emotional expressions and interactions affect mental health outcomes could inform the design of interventions aimed at enhancing well-being and treatment adherence in these communities. Furthermore, conducting separate analyses for each forum and comparative analyses across different time periods could offer deeper insights into user group patterns and changes over time. Exploring the identification of the most discriminating emotions in Part 1 would also enhance the depth of our analysis. Lastly, the integration of qualitative analyses to complement and validate findings derived from stylometry analyses could add a richer interpretative layer to the identified linguistic patterns, particularly in understanding nuanced contextual meanings.

## Conclusion

The findings of this study provide novel insights into the distinct characteristics of peer support user groups in online mental health forums. By conducting stylometry analysis on their written forum posts, we explored different types of stylometric features and found that the emotion feature type is particularly significant for distinguishing the user groups and predicting their writing styles. This suggests that emotional expression plays a crucial role in how users of these online communities communicate and interact with each other. In addition, this study comprehensively examined the differences of emotion expressions of the user groups to identify their similarities and differences. By doing so, we uncovered the emotional dynamics of these groups and how emotional expression is used to convey social support and promote well-being. These findings have important implications for developing community guidelines and strategies to manage and moderate emotional expression within these groups. By understanding how user groups interact with each other using emotional information, we can also develop more effective interventions and improve the quality of peer support offered within similar mental health communities. In conclusion, this study highlights the potential of emotional expressions to improve mental health in online communities. By taking a closer look at how users express and respond to emotions in these settings, we can develop better interventions and support structures that are tailored to the unique needs of different user groups. We hope that our findings will be useful to mental health professionals, community managers, and researchers in this field, and that they will contribute to a more comprehensive understanding of the emotional dynamics of online communities.

## Data Availability

The data that support the findings of this study are not openly available due to reasons of privacy and ethical considerations. Data are, however, available from the corresponding author upon reasonable request and with permission of the Swinburne University Human Research Ethics Committee and SANE.
